# Association between previous cataract surgery and cognition among middle-aged and older Chinese: the China health and retirement longitudinal study (CHARLS)

**DOI:** 10.1186/s12886-023-02998-y

**Published:** 2023-05-31

**Authors:** Xiaohuan Zhao, Kunchen Wei, Junran Sun, Jieqiong Chen, Yimin Wang, Yuhong Chen, Xinyue Zhu, Xiaodong Sun, Tong Li, Minwen Zhou

**Affiliations:** 1 Department of Ophthalmology, Shanghai General Hospital, Shanghai First People’s Hospital), Shanghai Jiao Tong University School of Medicine, Shanghai, China; 2 National Clinical Research Center for Eye Diseases, Shanghai, China; 3 Shanghai Key Laboratory of Fundus Diseases, Shanghai, China; 4 Department of Plastic and Reconstructive Surgery, Renji Hospital, School of Medicine, Shanghai Jiao Tong University, Shanghai, China

**Keywords:** Cataract surgery, Cognition, Chinese, CHARLS

## Abstract

**Background:**

Cataract is the primary cause of blindness globally, and surgery offers the only method by which to remove cataracts. We aimed to examine whether previous cataract surgery is associated with cognitive function.

**Methods:**

Our study included 13,824 participants. Data from the baseline of the China Health and Retirement Longitudinal Study (CHARLS) were used. The participants were categorized into two groups: with and without previous cataract surgery. Weighted multiple linear regression was used to obtain the β and 95% confidence intervals (CI).

**Results:**

The participants who had previous cataract surgery (n = 261) scored lower in cognition, including both memory and mental state, than those without previous cataract surgery. After adjusting for socioeconomic factors and metabolic measures, a negative association was evident between previous cataract surgery and cognition (β = −0.647, 95% CI: −1.244, − 0.049). Furthermore, the participants who were older and female demonstrated a decline in cognition, while living in cities and having higher levels education were associated with higher cognition.

**Conclusions:**

Better cognitive function was associated with less previous cataract surgery or cataract occurrence. This suggests that a period of vision loss due to cataract leads to cognitive decline, however further studies are need to dissect the impact of vision loss and cataract surgery on cognitive decline.

## Background

The growing number of older adults has raised significant health concerns worldwide. It is estimated that over 30% of the Chinese population will be 60 years or older by 2050 [1], and 30% of the European population will be 65 years or older by 2060 [2]. With the increasing aging population, a large number of elderly people will be at high risk of cognitive impairment [3, 4]. Cognitive function refers to multiple mental abilities, including memory, perception, attention, language, visuoconstruction, and executive functioning [5]. Cognitive impairment leads to a series of social and economic problems, including difficulties with disease management, caregiver burden, and a loss of productivity [6]. China has become the fastest growing country in the world for patients with cognitive impairment, with an annual growth rate of over 0.36 million patients [7, 8]. It is estimated that the total number of patients with cognitive impairment will reach 48.68 million by 2060 [8]. The need to study and identify the risk factors of cognitive impairment is therefore urgent.

With the aging of the population and increased life expectancy, cataract continues to be a leading public health issue. Cataract, which is the opacification of the lens, is the primary cause of blindness globally [9]. Cataract is the leading cause of blindness in patients aged 4 years and older worldwide, and the global prevalence of cataract is estimated to be 2.3% [10, 11]. Cataract surgery remains one of the most common and successful procedures currently undertaken in many countries, and its benefits may extend beyond vision improvement [12]. Cataract surgery is recognized for substantially ameliorating the quality of life of patients, with gains in their social and emotional lives [13]. The rate of cataract surgery per million people per year has been continuously improved from about 83 in 1988 to 2205 in 2017 in China [14]. Previous studies have shown that vision loss is one of the risk factors for cognitive decline[15, 16]. Growing epidemiological evidence suggests that cataract surgery helps improve cognition to an extent [17–19], but some reports have been negative or inconsistent [20, 21]. Most previous studies have been carried out in Europe, and studies on Chinese population are rare. As the largest developing country in the world, China has a significant number of elderly people who suffer from cataract and cognitive impairment.

To address this gap, we focused on middle-aged and older Chinese people to investigate the association between previous cataract surgery and cognition using data from the China Health and Retirement Longitudinal Study (CHARLS). In this study, cataract surgery is used as an indication that individuals experienced significant vision problems caused by cataracts, leading them to undergo surgery.

## Methods

### Study data

The data for this cross-sectional study were obtained from the CHARLS baseline dataset. The CHARLS is a nationally representative survey among middle-aged and older Chinese, and its baseline data cover 450 villages and 150 counties and districts across the country. The CHARLS baseline survey was carried out from June 2011 to March 2012 and included 17,705 subjects from 10,257 households. All the participants were interviewed one-on-one using a structured questionnaire, and their demographic, socioeconomic, lifestyle, and health-related data were recorded [22, 23]. The survey was approved by the Biomedical Ethics Review Committee of Peking University, and all data are available online at the CHARLS project website (http://charls.pku.edu.cn/).

Among 17,705 subjects at baseline, participants with missing data for age, gender, or cataract surgery were excluded from our study. We also excluded participants for whom residence, education or disease data (diabetes, dyslipidemia, or kidney disease) were missing. A total of 13,824 participants were included in our study.

### Assessment of cognitive function

Based on American health and retirement research, the participants’ cognitive function was assessed using the Telephone Interview of Cognitive Status (TICS) battery [24, 25]. Briefly, two aspects of cognitive function were evaluated, namely, memory and mental status. The subjects listened to 10 random irrelevant Chinese words and were then asked to recall them immediately (immediate recall; 0–10 points) and 4 minutes later (delayed recall; 0–10 points). Visuoconstruction was evaluated by copying a 5-edge figure (0–1 points), and mathematical capability was assessed by calculating 100 consecutively minus 7 (0–5 points). Orientation ability was evaluated by stating the current date, day of the week, and season (0–5 points). The total score for cognitive function was 31 points, and a higher score indicated better cognitive function.

### Assessment of previous cataract surgery

Previous cataract surgery was assessed by asking “Have you ever had cataract surgery?”. Based on the responses, we categorized the participants into two groups: with and without previous cataract surgery.

### Assessment of other variables

The participants’ age, place of residence (rural vs. urban), and level of education were self-reported. Education was divided into four levels: primary or below, middle school, high school, and college or above. Smoking status was assessed by asking “Have you ever chewed tobacco, smoked a pipe, smoked self-rolled cigarettes, or smoked cigarettes/cigars?”, and participants were divided into smoker and non-smoker groups. Drinking status were assessed by asking “Did you ever drink alcoholic beverages in the past? How often?”. Based on their responses, drinking status was set at three levels: drink more than once a month, drink less than once a month, and none of these.

For the present study, the participants’ personal health conditions were obtained by self-reporting via the question “Have you been diagnosed with hypertension, diabetes, and/or dyslipidemia by a doctor?”.

### Statistical analysis

The participants’ demographic characteristics were presented as mean ± standard deviation (SD) for the continuous variables and as percentages for the categorical variables. Taking into account the multistage sampling and nonresponse, we used weighted multiple linear regression to measure the association between previous cataract surgery and cognition. The model adjusted for age, sex, smoking status, drinking status, place of residence, education level, diabetes, hypertension, and dyslipidemia.

## Results

After filtering, 13,824 participants were deemed eligible for the current study. Among them, 261 participants had undergone previous cataract surgery. The average age of the previous cataract surgery group (69.36 ± 10.20 years) was higher than that of the without previous cataract surgery group (58.84 ± 9.99 years), and most of the participants in the previous cataract surgery group were women (62.1%). The participants in the previous cataract surgery group had a higher prevalence of hypertension (41.0%), diabetes (13.0%), and dyslipidemia (12.6%). The participants with previous cataract surgery were more likely not to drink and had a lower level of education. Furthermore, the participants in this group scored lower in terms of cognitive function, including both memory and mental state, than those without previous cataract surgery. The results of the statistical analyses of the participants’ demographic characteristics are shown in Table 1.

**Table 1:** Demographic characteristics of middle-aged and elderly Chinese with and without cataract surgery

Variables	Cataract surgery	Without cataract surgery	*P*
**Total**	261	13,563	
**Age**	69.36 ± 10.20	58.84 ± 9.99	< 0.001
**Sex, n (%)**			0.001
Male	99 (37.9)	6548 (48.3)	
Female	162 (62.1)	7015 (51.7)	
**Smoking status, n (%)**			0.593
Yes	162 (62.1)	5366 (39.6)	
No	99 (37.9)	8197 (60.4)	
**Drinking status, n (%)**			0.006
Drink more than once a month	45 (17.2)	3423 (25.2)	
Drink but less than once a month	18 (6.9)	1084 (8.0)	
None	198 (75.9)	9056 (66.8)	
**Hypertension, n (%)**			< 0.001
Yes	107 (41.0)	3272 (24.1)	
No	154 (59.0)	10,291 (75.9)	
**Diabetes, n (%)**			< 0.001
Yes	34 (13.0)	759 (5.6)	
No	227 (87.0)	12,804 (94.4)	
**Dyslipidemia, n (%)**			0.044
Yes	33 (12.6)	1224 (9.0)	
No	228 (87.4)	12,339 (91.0)	
**Residence, n (%)**			0.004
Urban	129 (49.4)	5492 (40.5)	
Rural	132 (50.6)	8071 (59.5)	
**Education, n (%)**			< 0.001
Primary or below	206 (78.9)	8926 (65.8)	
Middle school	29 (11.1)	2856 (21.1)	
High school	14 (5.4)	1426 (10.5)	
College or above	12 (4.6)	355 (2.6)	
**Cognition**	8.10 ± 5.19	9.98 ± 5.22	< 0.001
Memory	2.67 ± 2.12	3.32 ± 2.21	< 0.001
Mental status	5.43 ± 3.67	6.56 ± 3.64	< 0.001

To further clarify the association between previous cataract surgery and cognition, weighted multiple linear regression was applied, which are presented in Table 2. After further controlling for basic demographics, lifestyle habits, and health status, the cognitive score of the participants with previous cataract surgery remained 0.647 lower than that of the without previous cataract surgery group (β = −0.647, 95% CI: −1.244, − 0.049). For each year increase in age, there was a 0.166 decrease in mean cognitive score (β = −0.166, 95% CI: −0.182, − 0.149). Compared to the women, the mean cognitive score for the men was 0.906 points higher (β = 0.906, 95% CI: 0.741, 1.070). The participants who lived in cities scored on average 0.115 points higher in cognition than those in rural areas after controlling for socioeconomic and personal health conditions (β = 0.115, 95% CI: 0.099, 0.131). A higher educational level compared with a primary school-level education or below was associated with a mean higher cognitive score after adjusting for socioeconomic and personal health conditions. Participants with dyslipidemia showed 0.810 points higher in mean cognitive score than those without dyslipidemia (β = 0.810, 95% CI: 0.528, 1.092).

**Table 2:** Associations between previous cataract surgery and cognition in middle-aged and elderly Chinese (CHARLS 2011–2012)

	β (95% CI)	*P*
**Cataract surgery**	-0.647 (-1.244, -0.049)	0.034
**Age**	-0.166 (-0.182, -0.149)	< 0.001
**Sex**	0.906 (0.741, 1.070)	< 0.001
**Smoking status**	-0.013 (-0.034, 0.008)	0.218
**Drinking status**		
Drink more than once a month	ref	
Drink but less than once a month	-0.006 (-0.023, 0.001)	0.506
None of these	0.007 (-0.013, 0.026)	0.498
**Residence**	0.115 (0.099, 0.131)	< 0.001
**Education**		
Primary or below	ref	
Middle school	0.187 (0.171, 0.204)	< 0.001
High school	0.167 (0.150, 0.183)	< 0.001
College or above	0.109 (0.09, 0.125)	< 0.001
**Diabetes**	-0.013 (-0.028, 0.003)	0.124
**Hypertension**	0.002 (-0.014, 0.018)	0.816
**Dyslipidemia**	0.810 (0.528, 1.092)	< 0.001

## Discussion

In this cross-sectional study, we examined the association between previous cataract surgery and cognition among middle-aged and older Chinese. After adjusting for basic demographics, lifestyle habits, and health status, we found a negative association between previous cataract surgery and cognition. Our findings showed that previous cataract surgery was associated with a greater decline in cognitive function among the study population.

Notably, as this was a cross-sectional study, we used previous cataract surgery to represent cataract occurrence [20]. In other words, the results of the present study suggest that cataract occurrence, the leading cause of vision impairment, is significantly associated with cognition decline. Our results are in keeping with the findings of previous observational studies of older people with vision impairment showing a reduction in cognitive scores. In an older Malaysian population, vision impairment was associated with a significant decline in cognition after adjustment for age and sex (β = -1.863; 95% CI, -3.209, -1.081) [26]. After adjusting for potential confounding factors, 18% of adults aged ≥ 45 years with vision impairment in the United States reported subjective functional limitations related to cognitive decline compared to only 4% of those without vision impairment [27]. Based on the Health ABC study, visual acuity, contrast sensitivity, and stereo acuity impairments were associated with greater declines in cognitive functional scores over a 9-year period [28]. Compared with previous studies, our study focused on a middle-aged and older Chinese population whose cognitive function may have decreased slightly and in whom vision impairment caused by cataracts may appear in the near future. The data we used were from a nationally representative survey, which means the results of our study were generalizable to all middle-aged and older Chinese.

Some studies focused on older people who had cataract surgery with relatively normal cognition have shown a small but significant improvement in cognitive scores after surgery. Participants aged 75 years and older in northeast England showed significant improvements in their scores for Addenbrooke’s Cognitive Examination – Revised 1 year postoperatively [17]. In the Fujiwara-kyo Eye Study, cataract surgery reduced the risk of developing mild cognitive impairment but not dementia in subjects aged ≥ 68 years [19]. These longitudinal studies compared the participants’ cognitive levels before cataract surgery with those after cataract surgery while our study compared the cognitive function in people who underwent cataract surgery with that of people without cataract surgery in a relatively healthy large population. Our results found that previous cataract surgery was associated with a decline in cognitive scores, which suggests that severe visual impairment requiring cataract surgery is associated with decreased cognitive function.

There are some possible reasons for lower cognition among elderly with cataract occurrence. First, visual impairment due to cataract may contribute to difficulty in communication and social isolation, which may increase the risk of decline in cognitive function [29]. Secondly, there are some common risk factors between cataract occurrence and cognition decline, such as microangiopathy and older age, which may contribute to cognitive decline [30–32]. Furthermore, vision impairment due to cataracts reduces the external stimulation to central nervous system, which can lead to brain atrophy and decreased cognitive function [33, 34]. Finally, cataract surgery itself may result in decreased cognitive function due to anesthesia risks, as well as postoperative complications such as corneal endothelial decompensation, resulting in decreased vision [35].

In our study, socioeconomic factors and metabolic measures were considered potential cognitive confounders. We found that being elderly, being female, living in a rural area and having a low education level were associated with a more severe cognitive decline. It is common knowledge that age is one of the most important risk factors for cognitive impairment [36, 37]. Women account for two thirds of Alzheimer’s disease cases, which is consistent with the results of our study [38]. Sex hormones are currently used to explain gender differences in cognitive impairment, but the therapeutic effect of sex hormones on cognitive decline remains inconclusive [39, 40]. Compared with urban populations, people in rural areas have worse cognitive function. This may be explained by the availability of better medical and educational resources in cities. People with low levels of education also demonstrated low cognitive scores. Given the effects of education on cognitive impairment [41, 42], better cognitive function could be achieved through marked improvements in educational attainment.

The strength of the study lies in a large number of participants, setting it apart from previous studies and enhancing its credibility. However, our study had some limitations that should be noted. First, as a cross-sectional analysis, the time sequence of cataract occurrence and cognitive impairment was unclear. Further longitudinal studies are needed to confirm our findings. Second, even though we adjusted for a series of potential confounders, namely, basic demographics, lifestyle habits, and health status, some confounders were not considered or measured. Age is the most important risk factor for cataract, and trauma and heredity can also lead to cataract, none of which were evaluated in this study. Then, it is not accurate to use cataract surgery to represent cataract occurrence, although previous study has shown its feasibility [20].

## Conclusions

In conclusion, our study corroborated that less previous cataract surgery (cataract occurrence) is associated with better cognitive function among middle-aged and older Chinese. Being elderly, being female, living in a rural setting, and having a low education level were found to be associated with more severe cognitive impairment. Public health policies and programs, including timely interventions for diseases such as cataracts are therefore urgently needed.

## Data Availability

All methods were carried out in accordance with relevant guidelines and regulations or Declaration of Helsinki. The survey was approved by the Biomedical Ethics Review Committee of Peking University, and all data are available online at the CHARLS project website (http://charls.pku.edu.cn/).
